# Education Research: A Long-term Faculty Development Initiative Improves Specificity and Usefulness of Narrative Evaluations of Clerkship Students

**DOI:** 10.1212/NE9.0000000000200003

**Published:** 2022-09-22

**Authors:** Christopher J. Mooney, Stephen Joseph Powell, Spencer Dahl, Carly Eiduson, Benjamin Reinhardt, Robert Thompson Stone

**Affiliations:** From the Departments of Medicine (C.J.M.), and Neurology (S.J.P., B.R., R.T.S.), and Offices for Medical Education (S.D., C.E.), University of Rochester School of Medicine and Dentistry, NY.

## Abstract

**Background and Objectives:**

Narrative-based evaluations are increasingly used to discriminate between levels of trainee performance, yet barriers to high-quality narratives remain. Prior evidence shows mixed results regarding the effectiveness of faculty development efforts on improving narrative evaluation quality.

**Methods:**

We used a quasi-experimental study incorporating a historical control group to examine the effectiveness of a pragmatic, multipronged, 4-year faculty development initiative on narrative evaluation quality in a neurology clerkship. We evaluated narrative evaluation quality using the narrative evaluation quality instrument (NEQI) in random samples of narrative evaluations from a historical control and intervention group. We used multilevel modeling to compare NEQI scores (and subscale scores) across groups. Informed by the theory of deliberate practice, our faculty development initiative included (1) annual grand rounds sessions focused on developing high-quality narratives and reporting evaluation metrics, (2) restructuring the clerkship assessment form to simplify and prioritize narratives, (3) recruiting key faculty to rotate on the clerkship grading committee to gain experience with and practice developing quality narratives, and (4) instituting a narrative evaluation excellence award to faculty and residents.

**Results:**

The faculty development initiative was associated with improvements in the quality of students' narrative evaluations. Specifically, the intervention group was a significant predictor of NEQI score, with means of 6.4 (95% CI 5.9–6.9) and 7.6 (95% CI 7.2–8.1) for the historical control and intervention groups, respectively. In addition, the intervention group was associated with significant improvement in the specificity and usefulness NEQI subscale scores, but not the performance domain subscale score.

**Discussion:**

A long-term, multipronged faculty development initiative can facilitate improvements in narrative evaluation quality. We attribute these findings to 2 factors: (1) pragmatic, solution-oriented efforts that balance focused didactics with programmatic shifts that promote deliberate practice and skill improvement and (2) departmental resources that prioritize and convey a commitment to improving trainee assessment.

Valid assessment of clinical competence is an elusive and evolving exercise.^1-3^ Over the past several decades, assessment frameworks in medical education have moved beyond a singular focus on objectivity and minimizing human judgment toward paradigms that embrace more subjective and nonstandardized performance assessments to inform defensible decision-making of learner competence.^4-6^ Indeed, a growing body of literature now accepts that performance is socially constructed—conceptualized and negotiated by individual, situational, and environmental contexts—and the pursuit of a single truth or bias-free objectivity is a naive assumption and likely a fool's errand.^5,7-9^ Furthermore, the literature has established validity evidence of narrative assessments^4,10,11^ indicating that, relative to numeric-based assessments, constructivist-interpretivist assessment approaches provide meaningful^7,12,13^ and potentially more valid representations of trainee performance.^7,14^

Despite their advantages, the promise of narrative-based assessment is reliant on accurate and insightful comments. Yet, narrative evaluations are regularly perceived as vague,^15^ nonspecific,^16,17^ and prone to writers' idiosyncrasies that can impair interpretation.^18^ Studies also suggest that narratives are more often praising than critical,^17,18^ implicating a culture of politeness in medical education that impedes meaningful feedback.^18-21^ Reports of faculty development initiatives to generate higher-quality narratives are relatively limited and show mixed results. A study by Dudek et al.,^22^ for instance, found that a workshop to improve quality of in-training evaluation reports (ITERs) increased scores on the completed clinical evaluation report rating (CCERR) in a self-selected sample of 22 physicians; however, the small sample and absence of a control group preclude definitive conclusions of the intervention's efficacy. Conversely, a study of a similar faculty development workshop in pharmacology clinical supervisors failed to improve CCERR scores relative to historical controls.^23^ Beyond didactic workshops, efforts to alter the assessment environment including structural changes to ITER forms^24^ and increased continuity of supervision^25^ have proven similarly challenging to producing high-quality trainee assessments and speak to the task's difficulty more generally.

Notwithstanding the disparate evidence, targeted feedback and intervention has been shown to be an effective element to improving faculty teaching effectiveness^26^ and quality of rater-based assessments.^27^ With respect to the latter, Dudek et al.^27^ found that relative to controls, CCERR scores improved in an intervention group that received feedback on ITER quality over a 6-month period across several institutions, although the effect was relatively modest and the intervention was not specifically directed toward improving narrative comments.

The importance of feedback in promoting expertise is a guiding principle of the Ericson model of deliberate practice which necessitates deliberate reflection of feedback and subsequent practice.^28^ After calls to encourage rich, insightful, and accurate narratives^4,11,18,21,24^ and guided by tenets of deliberate practice,^28^ we sought to build upon the work by Dudek et al.^27^ and examined the extent to which a multipronged faculty development effort could improve the quality of medical students' narrative evaluations. We hypothesized that such an effort would demonstrate higher-quality narratives in the intervention group compared with a historical control group, as measured by the narrative evaluation quality instrument (NEQI), which comprehensively assesses the quality of narrative evaluations.^29^ As a secondary aim, we sought to collect additional validity evidence^30^ of the NEQI.

The findings from this study can advance understanding on how to improve and measure the quality of narrative evaluations, which is imperative given transitions to entrustment ratings and programmatic assessment models that are apt to increase the volume and reliance on narrative assessment of trainees.^7,31,32^ In addition, the recent elimination of the United States Medical Licensing Examination Step 2 Clinical Skills examination and increasing prominence of pass/fail grading schemes are likely to further increase the importance of narrative assessments because residency programs look for alternative means to discriminate between levels of trainee performance.^33^

## Methods

### Intervention

**Figure 1:**
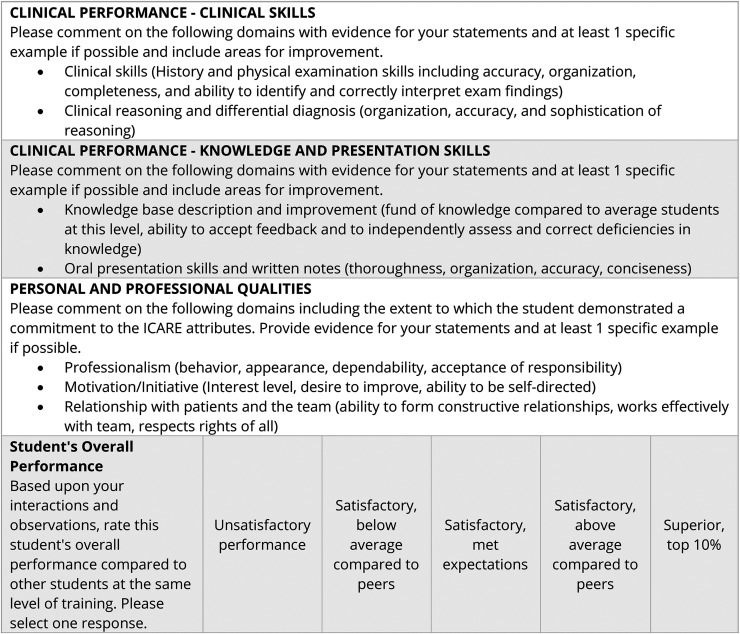
Neurology Clerkship Evaluation Form

We used a quasi-experimental study incorporating a historical control group to examine the effect of department-wide faculty development efforts around narrative evaluation quality over a 4-year period. Our faculty development efforts included a multipronged approach grounded in pragmatic, solution-oriented actions and included 4 key elements. First, we instituted an annual medical education grand rounds session focused on teaching core components of high-quality narrative assessment (e.g., use of examples and constructive critique) and reporting narrative evaluation quality metrics to faculty, residents, and other key stakeholders. The session encouraged participants to reflect upon and examine their own narrative assessments. We chose a grand rounds setting for this didactic session given high attendance, of which approximately 50% constitute department faculty and 25% residents. Second, we restructured the clerkship ITER form (Figure 1) to reduce item redundancy and evaluators' cognitive load. Specific efforts included the prioritization of narratives by removing all numeric scores and encouraging specific examples around learner performance. Third, we recruited key faculty who provide the largest quantity of trainee evaluations (e.g., neurohospitalists) to rotate on the neurology grading committee for a period of 1–2 years to familiarize them with quality narratives that can inform student assessment. The 7 members of the grading committee, including a rotating neurohospitalist, account for approximately 8% of all completed student ITERs. Last, we instituted an annual evaluation excellence award to select faculty and residents who consistently provided high-quality narrative evaluations as a means of recognizing outstanding evaluators. This laudatory award, selected by using a consensus process by the clerkship grading committee, is provided to 1–2 faculty and residents and announced at subsequent department grand rounds.

### Setting and Participants

The neurology clerkship at the University of Rochester School of Medicine and Dentistry is a 4-week inpatient rotation in the third year. All faculty spend 1 week with each student, with the exception of child neurologists who spend 2 weeks with students. Attending rotations are 1–2 weeks long. Residents typically rotate with an individual student for 2 weeks on a given service and are responsible for assigning students' patients, teaching on rounds, assigning and overseeing tasks, and providing on-the-fly and formal feedback. A request to evaluate students using a standardized ITER form is sent to faculty and residents after their scheduled rotation(s). Students generally receive between 5 and 9 ITERs. Narrative information from the ITERs accounts for 60% of students' grades.

We abstracted deidentified neurology clerkship narrative evaluations from 20 randomly selected medical students from the 2020–2021 academic year, resulting in a total of 118 unique narratives for the intervention group. We selected 20 medical students to provide an equivalent sample to the historical control group (n = 20) that were randomly selected during the 2016–2017 academic year and used in a previous study examining reliability evidence of a tool (below) to assess narrative quality,^29^ resulting in 123 unique control group narratives.

### Measures

**Figure 2:**
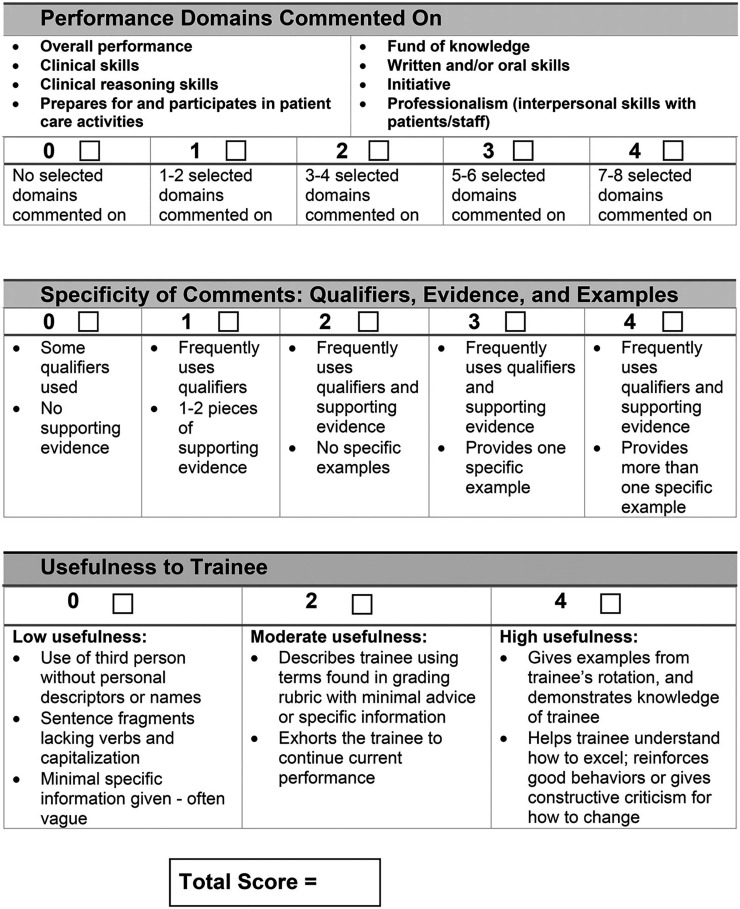
Narrative Evaluation Quality Instrument

We measured narrative evaluation quality in the historical control and intervention groups using the NEQI (Figure 2). The NEQI assesses the quality of narrative evaluations along several dimensions including performance domains, specificity of comments, and usefulness to trainees and has been shown to reliably differentiate between narrative quality.^29^ Before narrative evaluation review, 4 reviewers (authors: S.J.P., S.D., C.E., and B.R.) used the NEQI training guide alongside 12 student evaluations for training purposes. Once consistency was established, the 4 reviewers independently assessed each of the remaining 20 students' total narrative evaluations (n = 118), which comprised the analytic sample for the intervention group. We then compared NEQI scores of this cohort with the historical controls' NEQI scores (n = 123), which were assessed by 5 different reviewers.

### Analysis

We used linear mixed-effects modeling to compare NEQI scores across the control and intervention groups. Mixed-effects modeling allows for the partitioning of variance components and is appropriate for clustered data, such as repeated observations across students and faculty and nested data structures (e.g., students within faculty).^34^ We began by fitting an unconditional model to estimate the proportion of variance because of clustering between students and faculty and to confirm the appropriateness of a mixed-model analysis. We then developed a random intercept model to predict NEQI score, including study group (historical control or intervention) and reviewer as fixed effects and allowing the intercepts to vary across faculty (level 2) and student (level 3). We estimated reliability for total NEQI scores with intraclass correlations coefficients (ICCs). In secondary analyses, we used multilevel generalized linear models to compare the NEQI subscale scores (e.g., domain, specificity, and usefulness) across the historical control and intervention groups. Multilevel generalized linear models were used given their flexibility with potentially nonlinear responses that may exist given few response options within the NEQI subscales.^35^ We also compared the average count of each performance domain (e.g., clinical reasoning and fund of knowledge) across groups. We observed no missing data in study variables. We used Stata/SE version 14.2 (College Station, TX) for all analyses.

### Ethics

The University of Rochester Medical Center's Research Subjects Review Board approved this study.

### Data Availability

Study data supporting the findings are available upon request after review and approval by the University of Rochester's Research Subjects Review Board.

## Results

For the intervention group, 4 reviewers assessed a total of 118 unique narrative evaluations of students, resulting in 472 NEQI scores. The intervention group's narratives were composed of 55 assessors (60.0% attendings; 40.0% residents), with an average of 2.1 (SD = 1.3) narratives per assessor. In the historical control group, 5 reviewers assessed 123 unique narrative evaluations, resulting in 615 NEQI scores. The control group's narratives were composed of 53 assessors (62.3% attendings and 37.7% residents), with an average of 2.3 (SD = 1.5) narratives per assessor. The range of narratives completed across groups was similar (1–6). Examination of participants indicated that 17 assessors were in both the control and intervention groups, resulting in 91 unique assessors in the study sample.

A likelihood-ratio test comparing the fit of the unconditional model with a conventional linear model confirmed a significant improvement in model fit (χ^2^(2) = 1,098.5, *p* < 0.0001), thus warranting a mixed-model analysis. Analysis of the unconditional models across study group revealed NEQI grand means of 6.4 (95% CI 5.90–6.90) and 7.6 (95% CI 7.15–8.10) for the historical control and intervention groups, respectively. Examination of interrater reliability revealed relatively similar ICCs across the 2 groups (ICC intervention group: 0.81 [95% CI 0.76–0.85]; ICC control group: 0.79 [95% CI 0.74–0.84]).

The subsequent development of the random intercept model including reviewer and cohort as fixed effects revealed a significant improvement in model fit when compared with the unconditional model with a likelihood-ratio test (χ^2^(8) = 103.39, *p* < 0.0001). Examination of model estimates indicated that study group was a significant predictor of NEQI scores (*b* = 1.58, *p* < 0.0001), controlling for reviewer effects (Table). With respect to the analysis of NEQI subscales, generalized linear models further revealed that study group was a statistically significant predictor of specificity (*b* = 1.01, *p* < 0.0001) and usefulness (*b* = 0.45, *p* = 0.005) subscales. Conversely, study group was not a significant predictor for performance domain subscale score (*b* = 0.13, *p* = 0.241). The results of models and means of NEQI subscale scores across groups are presented in Table.

**Table:** Mean Total and Subscale NEQI Scores for Control and Intervention Groups and Parameter Estimates for Effect of Intervention Group on Total and Subscale NEQI Scores

Group	NEQI Total Mean (95% CI)	NEQI Performance domains Mean (95% CI)	NEQI Specificity Mean (95% CI)	NEQI Usefulness Mean (95% CI)
Intervention	7.6 (7.2–8.1)	2.99 (2.92–3.01)	2.22 (2.12–2.33)	2.40 (2.30–2.51)
Control	6.4 (5.9–6.9)	2.61 (2.53–2.68)	1.81 (1.72–1.89)	1.96 (1.85–2.07)

NEQI measure^a^	*B* (95% CI)	SE	*p* Value	
Total score^b^	1.58 (0.90 to 2.27)	0.35	<0.0001	
Performance domains^c^	0.12 (−0.08 to 0.32)	0.10	0.24	
Specificity^c^	1.01 (0.74 to 1.27)	0.14	<0.0001	
Usefulness^c^	0.45 (0.14 to 0.77)	0.16	0.005	

Abbreviation: NEQI = narrative evaluation quality instrument.

a Only model fixed effects for the effect of the intervention group are displayed.

b Estimated using mixed-effects modeling.

c Estimated using generalized linear modeling.

Examination of performance domain counts across the 2 groups yielded notable findings. With the exception of the overall performance domain that was greater in the control group (χ^2^(1) = 452.6, *p* < 0.0001), the intervention group had a greater number of narratives commenting on students' clinical reasoning (χ^2^(1) = 274.8 *p* < 0.0001), fund of knowledge (χ^2^(1) = 34.1 *p* < 0.0001), clinical skills (χ^2^(1) = 98.3 *p* < 0.0001), preparation/participation in care (χ^2^(1) = 7.3 *p* = 0.007), written/oral presentation skills (χ^2^(1) = 52.5 *p* < 0.0001), and professionalism (χ^2^(1) = 41.1 *p* < 0.0001). There were no differences in the initiative performance domain across groups (χ^2^(1) = 1.67 *p* = 0.197).

## Discussion

Narrative-based assessment has emerged as a critical component of clinical assessment, yet barriers to high-quality narratives remain. In this study, we aimed to compare the quality of faculty narrative evaluations after implementation of a multipronged, 4-year faculty development program. Using a historical control design, we found evidence that our pragmatic faculty development efforts, which focused on developing high-quality narratives, were associated with a higher quality of medical student narrative evaluations. Specifically, assessors in the intervention cohort had significantly higher NEQI scores relative to historical controls.

The finding that our intervention increased the specificity and usefulness NEQI subscale scores, but not the performance domain subscale, is important. A subsequent analysis comparing counts of performance domains across cohorts indicated that the intervention group commented less on students' overall performance but had an increased number of comments on all but one specific performance domain. These findings suggest that assessors were providing more detailed descriptions of trainee performance around specific clinical domains including the use of examples to substantiate written feedback and inform trainees' goal development; however, the scope of narratives remained relatively similar across groups. A lack of improvement with respect to the total number of performance domains commented on is noteworthy and could be explained by evaluation burden, assessor time limitations, or limits to working memory vis-a-vis extraneous cognitive load.^36^ Alternatively, Cheung et al.^25^ have suggested that assessors' judgments are influenced by gestalt impressions and relatively limited aspects of performance, which could explain a propensity to comment on a relatively limited number of performance domains.

Our finding of higher-quality narratives in the intervention group compares favorably with the broader literature, which has shown mixed results regarding the effect of faculty development efforts on narrative quality^22-25^ including negligible effects and self-selected samples, with the latter potentially favoring the inclusion of participants more invested in trainee evaluation.^37,38^ Distinct from prior studies,^22,23,27^ we procured students' narratives through a random selection process which is likely to provide a more heterogeneous sample with respect to assessors' educational interests and training, thus enhancing generalizability of results. With respect to the observed effect, the relative difference in the control and intervention groups might appear modest; however, our effect is similar in magnitude to prior studies that have shown improvements in assessments, including written comments, after rater/assessor training.^27,39,40^ In addition, our prior work developing the NEQI^29^ has suggested that an NEQI score of 7 represents a minimum quality threshold, with the bulk of evaluations in the historical control group failing to reach this level, although further work is needed to understand whether this threshold is of educational significance.

The study findings have important implications for narrative assessment and trainee evaluations more generally. Most notably, this study extends evidence suggesting that multipronged faculty development initiatives may be better positioned to facilitate improvements in narrative assessments of trainees relative to single, isolated interventions.^22,25,27^ Such interventions, particularly when incorporating process-level and structural-level changes to affect the broader learning environment and culture around trainee assessment, are poised to facilitate skill improvement and mastery. Although prior work with the NEQI has established several sources of validity evidence including that regarding content and internal structure,^14,29^ this study provides additional evidence relating to consequences^41^ by documenting the intended effect of the intervention through an improvement in the quality of narrative assessments. It is important that no unintended effects of the NEQI scores were observed based by removing numeric scores from the clerkship ITERs. The intersection of study findings with our guiding theoretical framework also warrants consideration. Our faculty development intervention was designed to foster structured activities (e.g., self-reflection, practice) to improve performance in a domain-specific area (e.g., narrative assessment), which is consistent with the original definition of deliberate practice.^42,43^ However, research scrutiny has suggested that conditions for deliberate practice—individualized training directed by a qualified teacher—are rarely met and instead may reflect other nuanced forms of practice including *purposeful* and *naive* practice, which may have differential effects on performance.^42,44^ A similar dispute centers on whether deliberate practice must be an independent activity or whether it can also comprise groups, such as faculty.^42,44^ Ultimately, the definitional confusion and evolving conceptualization of deliberate practice have led some to question whether researchers can consistently conceptualize, apply, and test this theory in their work.^45^ Although it is reasonable to ask whether our study meets the hallmarks of deliberate practice, we contend that flexibility is needed for theoretical praxis in complex, resource-constrained settings, such as faculty development, where periodic, individualized training across large cohorts is often impracticable. Instead, our findings suggest that a theoretically informed intervention focused on structural-level and process-level factors that support domain-specific practice can be readily applied and are linked with performance improvement. It is also arguable that such contexts may comprise a necessary condition for performance improvement beyond a general accounting of one's total accrued time engaged in (deliberate) practice activities—a claim supported by multifactorial models of expertise that consider expertise a product of multiple factors including environmental and experiential influences.^45^

Study conclusions should be qualified in light of limitations. First, this study focused on one department (neurology) within a single institution, which could affect the transferability of findings to other contexts. Although we have expanded our efforts to other learners in different settings locally, similar studies are needed to replicate effects beyond our own institution. Second, given the random selection process of student narratives, it is unclear whether all narrative authors received equivalent exposure to the intervention. Thus, our effect estimate may underestimate or overestimate the true effect of the intervention. Nevertheless, as previously mentioned, our findings are analogous in scope and direction to similar studies, which strengthens the transferability of findings.^27,39,40^ Third, as discussed, our study builds upon efforts documenting the effectiveness of multipronged faculty development efforts; however, the complex nature of such interventions hinders precise replication and the ability to identify specific elements that result in an educationally beneficial finding.^46^ Although theoretically and empirically informed interventions can help minimize these constraints,^46^ isolating a single causative agent may be an unreasonable expectation given the multifactorial nature of teaching and learning within complex educational systems. Relatedly, in the absence of a contemporaneous control group, it is possible that other concurrent changes in the educational program including maturation of assessors or inconsistency of assessors across groups, rather than the intervention itself, may be responsible for changes in scores across time.^47^ However, the random selection process of students' narratives and moderate consistency of assessors (approximately one-third of the intervention group) suggests that the effect of these threats is relatively minimal.

In addition, our study examined narrative quality and was not designed to assess narrative accuracy—both of which are critical to providing valid clinical assessment and entrustment decisions. Indeed, evidence has suggested that cultural, social, and organizational tendencies such as saving face may result in politeness strategies that impede authentic feedback and assessment.^20,48^ Relatedly, our intervention was focused solely on developing high-quality narratives and did not aim to alter specific aspects of the clinical learning environment (e.g., providing greater direct observation), which could potentially mediate high-quality feedback and assessment by optimizing the teacher-learner relationship.^20^ To this end, we echo the call of Cheung et al.^25^ that additional research is necessary to better understand how an educational alliance can positively affect each of these subcomponents and narrative quality more generally.

In conclusion, the findings from this study show that a pragmatic, multipronged faculty development initiative predicated on tenets of deliberate practice, which used the NEQI as a teaching and feedback mechanism, is associated with improvements in the quality of narrative evaluations of medical students. Departmental resources were critical to developing and embedding these efforts into our education program and conveying a collective commitment to improving trainee assessment. Although prior work with the NEQI has established several sources of validity evidence including content and internal structure,^14,29^ future work will collect examine consequences evidence^30^ by how examining trainees and promotion committees may differentially interpret and use higher-scored vs lower-scored comments using the NEQI. Future research will also involve identifying specific assessor-level factors that are associated with overall and subscale NEQI scores and examining the effect of providing individualized feedback, rather than aggregate group feedback, on narrative quality. Such efforts have the potential to inform more focused individual-level interventions around narrative assessment quality in health professions education.

## Appendix

**Appendix:** Authors

Name	Location	Contribution
Christopher J. Mooney, PhD, MPH, MA	Department of Medicine, University of Rochester School of Medicine and Dentistry, NY	Drafting/revision of the manuscript for content, including medical writing for content; study concept or design; analysis or interpretation of data
Stephen Joseph Powell, MD	Department of Neurology, University of Rochester School of Medicine and Dentistry, NY	Drafting/revision of the manuscript for content, including medical writing for content; analysis or interpretation of data
Spencer Dahl, BS	Offices for Medical Education, University of Rochester School of Medicine and Dentistry, NY	Drafting/revision of the manuscript for content, including medical writing for content; analysis or interpretation of data
Carly Eiduson, BS	Offices for Medical Education, University of Rochester School of Medicine and Dentistry, NY	Drafting/revision of the manuscript for content, including medical writing for content; analysis or interpretation of data
Benjamin Reinhardt, MD	Department of Neurology, University of Rochester School of Medicine and Dentistry, NY	Drafting/revision of the manuscript for content, including medical writing for content; analysis or interpretation of data
Robert Thompson Stone, MD	Department of Neurology, University of Rochester School of Medicine and Dentistry, NY	Drafting/revision of the manuscript for content, including medical writing for content; major role in the acquisition of data; study concept or design; analysis or interpretation of data

## Glossary

CCERR: completed clinical evaluation report rating
ICC: intraclass correlations coefficient
ITER: in-training evaluation report
NEQI: narrative evaluation quality instrument
